# The estimation of pubertal growth spurt parameters using the superimposition by translation and rotation model in Korean children and adolescents: a longitudinal cohort study

**DOI:** 10.3389/fped.2024.1372013

**Published:** 2024-09-19

**Authors:** Dohyun Chun, Seo Jung Kim, Yong Hyuk Kim, Junghwan Suh, Jihun Kim

**Affiliations:** ^1^ College of Business Administration, Kangwon National University, Gangwon-do, Republic of Korea; ^2^ Research Team, The Global Prediction Co., Ltd., Gyeonggi-do, Republic of Korea; ^3^ Department of Pediatrics, Severance Children’s Hospital, Yonsei University College of Medicine, Seoul, Republic of Korea; ^4^ Department of Pediatrics, Wonju Severance Christian Hospital, Gangwon-do, Republic of Korea; ^5^ College of Humanities & Social Sciences Convergence, Yonsei University, Gangwon-do, Republic of Korea

**Keywords:** height, growth curve, growth velocity, pubertal growth, growth spurt, SITAR

## Abstract

**Objectives:**

Understanding the characteristics of the pubertal growth spurt in Korean children and adolescents can serve as crucial foundational data for researching puberty and growth-related disorders. This study aims to estimate the key parameters of pubertal growth, specifically the age and magnitude of the pubertal growth spurt, utilizing longitudinal data from a cohort of Korean children and adolescents.

**Methods:**

This study used mixed longitudinal height data from a cohort of Korean elementary, middle, and high school students aged 7–18 years. The Superimposition by Translation and Rotation (SITAR) model, a shape-invariant growth curve model, was utilized to estimate a reference height velocity curve for the entire dataset and individual curves via random effects to evaluate pubertal growth parameters. Altogether, 3,339 height measurements (1,519 for boys and 1,820 for girls) from 270 individuals (123 boys and 147 girls) were analyzed.

**Results:**

The average age of growth spurt onset in Korean boys was 10.17 ± 0.61 years (mean ± SE), with peak height velocity occurring at 12.46 ± 0.69 years of age (9.61 ± 1.26 cm/year). Korean girls, contrarily, experience their growth spurt at an earlier age (8.57 ± 0.68 years), with peak height velocity occurring at 10.99 ± 0.74 years of age (8.32 ± 1.09 cm/year). An earlier onset of puberty in both sexes is associated with a shorter growth spurt duration (0.63 years for boys and 0.58 years for girls) and a higher peak height velocity (1.82 cm/year for boys and 1.39 cm/year for girls). These associations were statistically significant for both sexes (all *p* < 0.0001).

**Conclusion:**

This study is the first to use the height velocity curve from the SITAR model to examine the pubertal growth spurt of Korean children and adolescents. The estimated timing and magnitude of the pubertal growth spurt, and their relationships can be useful data for clinicians and researchers.

## Introduction

Puberty is a biological and psychological transition from childhood to adulthood, marked by significant changes in height, weight, and body composition (1, 2). In the pubertal phase, increased levels of sex hormones lead to an enhancement in growth hormone pulse intensity, triggering a growth spurt (3). Early pubertal changes, characterized by growth velocity acceleration and rapid bone maturation, frequently lead to a decrease in adult height. Conversely, delayed puberty is associated with a reduced or absent growth spurt, resulting in diminished adult height and decreased bone mineral density (4, 5). Therefore, studying the timely initiation of puberty and the corresponding appropriate pubertal growth is crucial for analyzing growth. Additionally, by understanding pubertal growth, growth velocity can be employed as a non-invasive parameter for assessing puberty onset and progression.

To assess for maturational status, peak height velocity and age of peak height velocity are frequently employed as non-invasive measures in growth observation studies (6). Age of peak height velocity refers to the age at which the maximum growth velocity occurs during puberty, which is approximately 2 and 1.5 years after puberty onset in boys and girls, respectively (7). Thus, pubertal timing is evaluated using a surrogate peak height velocity marker. The significance of the age of peak height velocity lies in its uniform applicability to both sexes and its objectivity, contrasting with traditional puberty determination methods, such as bone age imagery or Tanner staging. As a result, the age of peak height velocity is widely used as an outcome or covariate to control for confounding in studies related to puberty and growth (8–16). Nevertheless, the age of peak height velocity poses challenges, requiring continuous height growth monitoring until the pubertal growth spurt stops. Directly observing height velocity and acceleration is impractical, necessitating indirect derivation through height observations and subsequent computation of differences. There is uncertainty regarding whether an optimal model for height is equally effective for velocity and acceleration. To tackle these challenges, various linear and non-linear parametric models have been proposed (17–20).

Cole, Donaldson, and Ben-Shlomo proposed a shape-invariant model encompassing a single fitted curve, i.e., the Superimposition by Translation and Rotation (SITAR) model, to capture the shared attributes prevalent during childhood and adolescence (21). The SITAR model is a non-linear multilevel construct that incorporates natural cubic splines, enabling the estimation of the average growth curve for a population and distinguishing deviations from this average as random effects. A distinct aspect of this model is its ability to estimate the benchmark growth curve for the whole sample, and subsequently derive individual curves by fine-tuning specific parameters, also known as random parameters. These parameters are as follows: a size parameter, signifying mean height; a tempo parameter, indicative of timing; and a velocity parameter, reflecting compression and expansion. Consequently, these three key factors are posited to largely determine an individual's growth curve. By utilizing the estimated individual velocity curve, the SITAR model facilitates the identification of the growth spurt period, which is characterized by a rapid growth rate. It further enables the estimation of growth parameters, including the age of onset of the growth spurt, the onset of growth spurt velocity, and growth spurt interval, alongside age at peak height velocity and peak height velocity.

The present study aimed to establish a growth curve for pubertal growth among Korean children and adolescents and analyze the growth spurt parameters by applying the SITAR model to longitudinal growth data.

## Materials and methods

### Study sample

The Global Prediction Co., Ltd. (GP) Cohort Study is a mixed longitudinal investigation involving Korean students, which is an ongoing cohort conducted by Global Prediction Co., Ltd., Gwangmyeong City, Republic of Korea. GP is a company that conducts growth research based on children's biometric data. It has conducted various national projects related to child and adolescent growth and has obtained clinical Good Manufacturing Practices from the Ministry of Food and Drug Safety for its growth testing software. The cohort consists of elementary, middle, and high school students (aged 7–18 years) in South Korea, specifically in Gwangmyeong City, Gyeonggi Province, with an average of 35 schools participating annually. The data were collected by visiting schools at least twice a year, where examiners measured the height, weight, and body composition of all students using an octapolar multifrequency biometric impedance analyzer (Inbody models J10 and J30, Inbody, Seoul, Korea) according to a standard protocol. Participants who were eligible for physical measurements and body composition assessments were enrolled upon obtaining agreement from both guardians and participants. Individuals who were unable to undergo such measurements due to physical conditions or refused participation were excluded from the measurement targets. The study started on January 1, 2013, and, as of April 30, 2023, 588,546 data points on 96,485 children and adolescents (50,480 boys and 46,005 girls) who were born between 1998 and 2020 were accumulated. The present study used part of the GP Cohort data.

In this study, we applied the SITAR method to analyze pubertal growth spurts using GP cohort data. To enhance estimation performance, we followed the methodology of previous research, including studies by Cole, Donaldson, and Ben-Shlomo (21), and constructed the data subset for analysis as follows. Age intervals from 7 to 16 years were divided into five segments: 7 to <8.8 years, 8.8 to <10.6 years, 10.6 to <12.4 years, 12.4 to <14.2 years, and 14.2 to <16 years. Subsets were then created for boys and girls who had at least one observation in each interval. Participants lacking confirmed measurements at least once within the designated five age groups were excluded from the analysis. Exclusions encompassed instances where measurement data was lost, the measurement period did not comprehensively cover all age groups, or there were refusals to participate in certain measurement intervals. As a result, the dataset comprises 3,380 height measurements, with 1,519 for boys and 1,820 for girls, involving a cohort of 270 individuals (123 boys and 147 girls). On average, 12.4 height measurements were available per individual. This represents approximately 0.3% of the total participants.

### Data analysis

The SITAR methodology is a shape-invariant growth model employed to estimate a reference height velocity curve for the entire dataset, with individual curves being estimated by using three random parameters. The model is defined by the following equation:

(1) $$h_{it}=\alpha_{i}+g\left(\frac{t-\beta_{i}}{\exp(-\gamma_{i})}\right),$$

where *h_it_* represents the height of individual *i* at age *t*, g(t) is a natural cubic spline that models the average height-for-age relationship across all subjects. The parameters *α_i_*, *β_i_*, and *γ_i_* are subject-specific random effects that capture individual variations from the average growth pattern. Each parameter has a distinct interpretation:

• Size parameter (*α*): This adjusts the overall height level for each individual, effectively shifting the entire growth curve up or down. It accounts for differences in average height between subjects.
• Tempo parameter (*β*): This shifts the timing of the pubertal growth spurt along the age axis. It reflects individual differences in the onset of puberty and the age at which peak height velocity occurs.
• Velocity parameter (*γ*): This modifies the rate of growth during the pubertal period. It adjusts the shape of the growth curve by either compressing or stretching it along the age axis, thereby affecting both the duration and intensity of the growth spurt.

The results of each SITAR analysis yielded a comprehensive set of subject-specific size, tempo, and velocity parameters, supplemented by fixed effects for the average curve coefficients. These random effects were used to identify the pubertal growth spurt, which is a period of rapid increase and decrease in height velocity (22). The beginning of the growth spurt denoted as the age of onset of the growth spurt was defined as the point where the slope of the velocity curve rose most steeply. The growth velocity at this time was termed the onset of growth spurt velocity. The age and magnitude of peak height velocity were referred to as the age of peak height velocity and peak height velocity, respectively. The end of the growth spurt period was defined as the point where the height velocity became equal to the onset of the growth spurt velocity after the age of peak height velocity. The interval between the two periods was referred to as the growth spurt interval. To fit the SITAR model, we employed the SITAR package in R version 4.2.2.

### Statistical analysis

To provide a comprehensive representation of the distribution, pubertal growth parameters were summarized with both means and standard deviations, in addition to centile values (3%, 5%, 10%, 25%, 50%, 75%, 90%, 95%, and 97%). To assess the interrelationships among the pubertal growth parameters, we computed the Pearson correlation coefficients and their respective significance levels between the three SITAR random parameters characterizing the individual curves.

To further investigate the impact of these pubertal growth parameters, we categorized the dataset into the following two groups based on each parameter: the top 20% sample and the bottom 20% sample. We then conducted independent two-sample *t*-tests to evaluate the potential mean differences in pubertal growth parameters between these two groups. This approach allowed us to quantify how variations in one growth parameter correspond to differences in others, providing insights into their interrelationships. Specifically, this analysis enabled us to examine how earlier or later occurrence of one parameter (e.g., Age of peak height velocity) relates to changes in others (e.g., Growth spurt interval and peak height velocity), and to identify significant patterns such as the negative relationship between peak height velocity and growth spurt interval across both sexes.

The age of onset of the growth spurt, age of peak height velocity, and growth spurt interval were measured in years, whereas the onset of growth spurt velocity and peak height velocity were expressed in cm/year. All statistical analyses were performed using Python version 3.9.7 and R version 4.2.2. *P* ≤ 0.05 was considered statistically significant.

## Results

### Height and velocity trajectories

**Figure 1:**
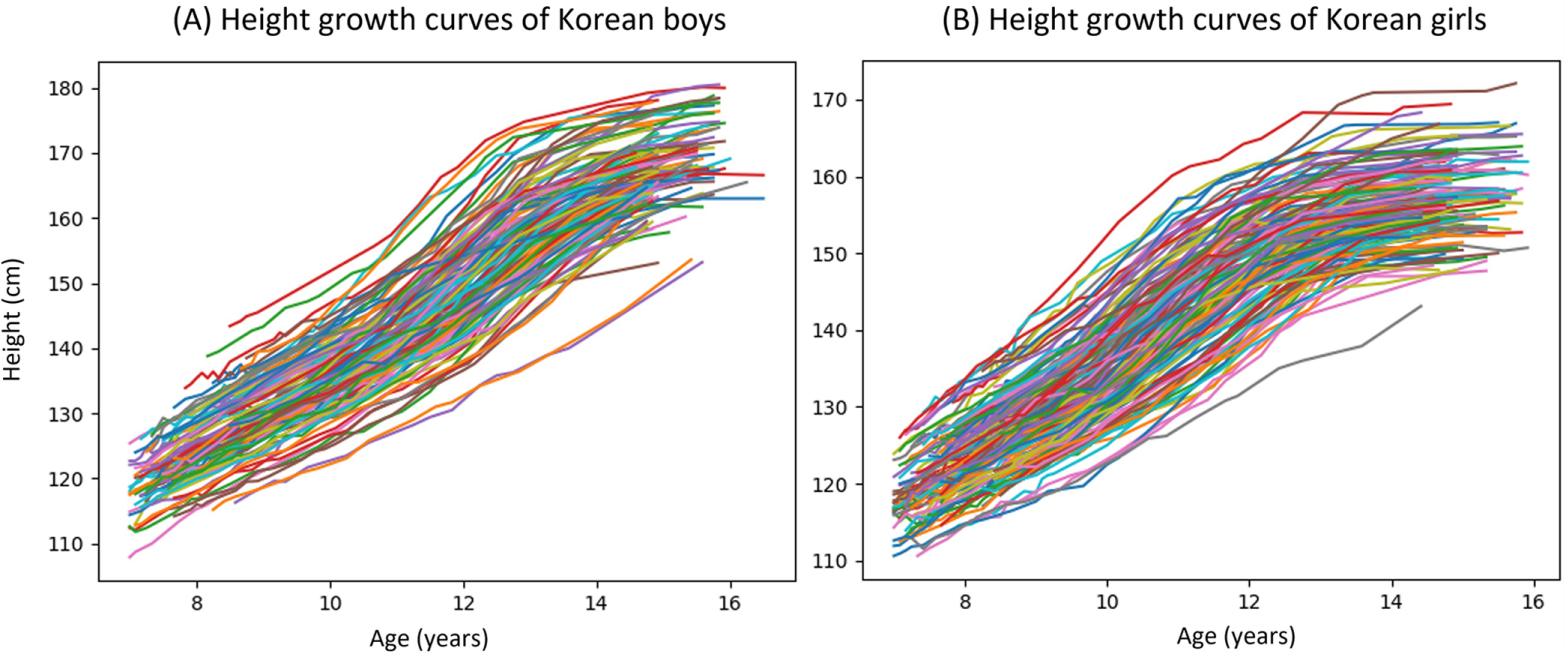
Height growth curves of Korean adolescents. **(A)** Height growth curves of Korean boys — 123 curves. **(B)** Height growth curves of Korean girls — 147 curves.

**Figure 2:**
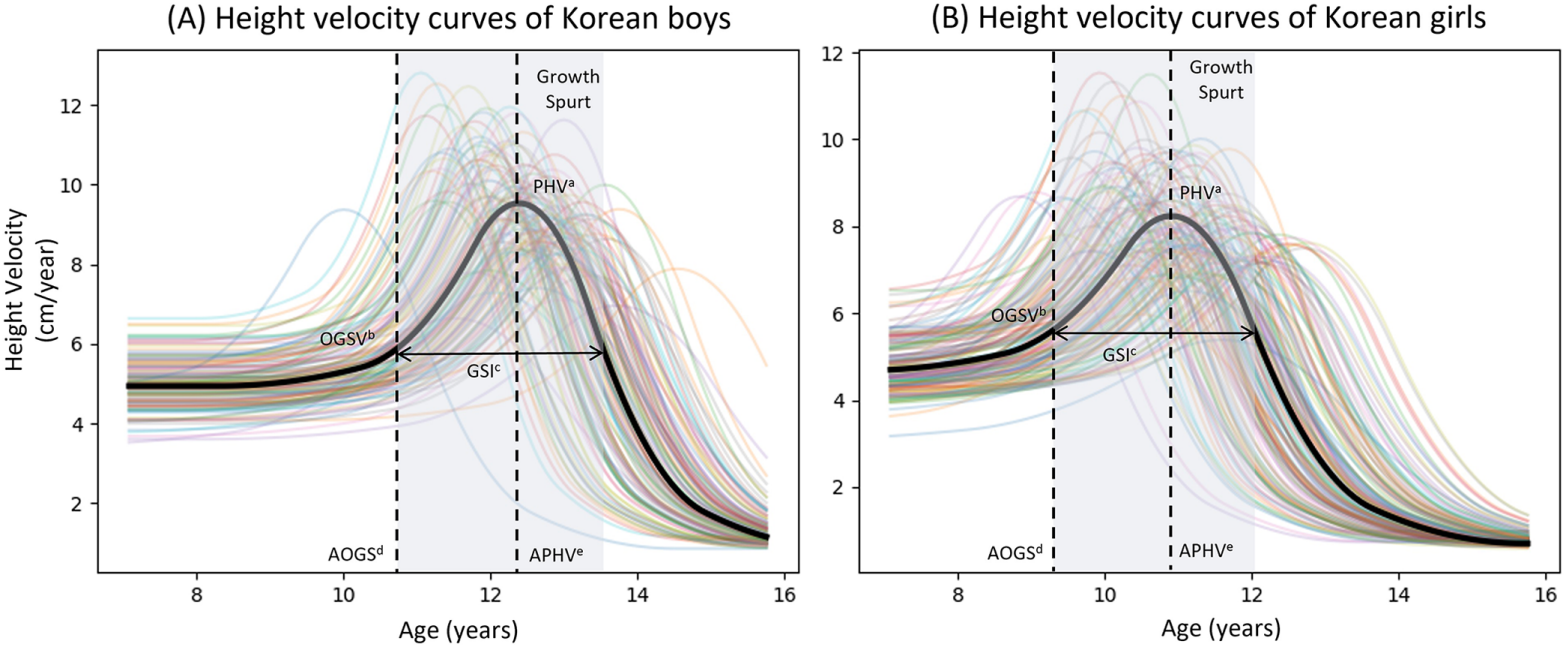
Mean and individual height velocity curves using the SITAR method. **(A)** Height velocity curves of Korean boys. **(B)** Height velocity curves of Korean girls. ^a^PHV, peak height velocity. ^b^OGSV, onset of growth spurt velocity. ^c^GSI, growth spurt interval. ^d^AOGS, age of onset of growth spurt. ^e^APHV, age of peak height velocity.

In Figure 1, the raw data obtained from the boys and girls in the GP Cohort are displayed, presenting a series of superimposed growth curves that are color-coded to distinguish individual subjects. Figure 2 depicts the average and individual height velocity curves derived from the SITAR estimation. The pubertal growth spurt, shown by the shaded area, along with the corresponding pubertal growth parameters, including age of onset of growth spurt, onset of growth spurt velocity, age of peak height velocity, peak height velocity, and growth spurt interval are also displayed. The mean absolute errors of SITAR estimations were 0.62 and 0.57 cm for boys and girls, respectively. Notably, individuals exhibited considerable variability in terms of the timing and magnitude of their pubertal growth. On average, boys tended to experience peak velocity at a later age, with greater magnitudes as compared to girls.

### Estimates of the timing and magnitude of the pubertal growth

**Figure 3:**
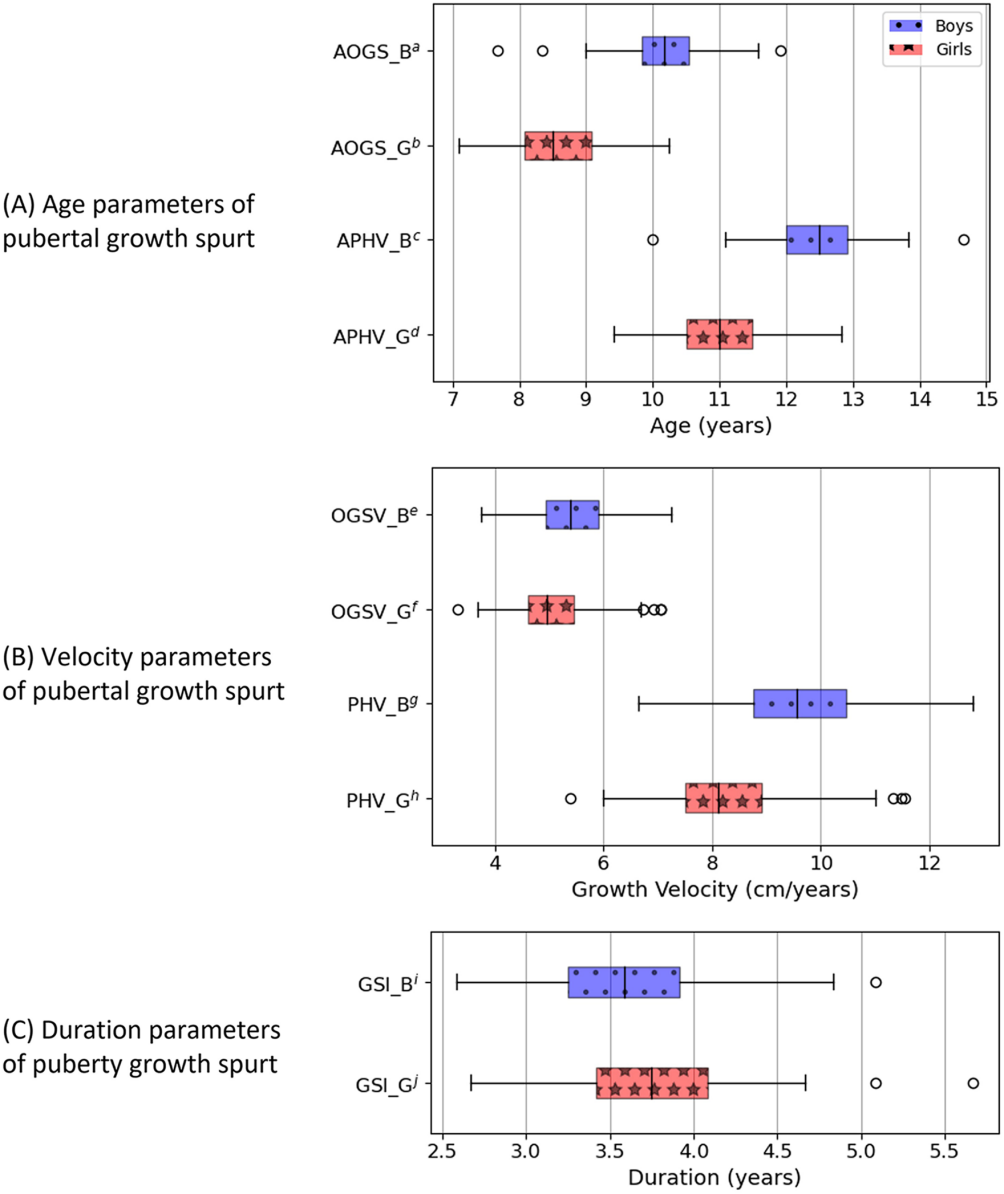
Boxplots for pubertal growth parameters. **(A)** Age parameters of pubertal growth spurt. **(B)** Velocity parameters of pubertal growth spurt. **(C)** Duration parameters of puberty growth spurt. ^b^AOGS_G, age of onset of growth spurt for girls. ^c^APHV_B, age of peak height velocity for boys. ^d^APHV_G, age of peak height velocity for girls. ^e^OGSV_B, onset of growth spurt velocity for boys. ^f^OGSV_G, onset of growth spurt velocity for girls. ^g^PHV_B, peak height velocity for boys. ^h^PHV_G, peak height velocity for girls. ^i^GSI_B, growth spurt interval for boys. ^j^GSI_G, growth spurt interval for girls.

As shown in Table 1, a statistically significant difference was observed in the timing of the pubertal growth spurt between the girls and boys. On average, boys reached the age of onset of the growth spurt and age of peak height velocity at 10.17 ± 0.61 and 12.46 ± 0.69 years, respectively, indicating that peak height velocity occurs approximately 2.29 years after the commencement of the pubertal growth spurt in boys. Contrarily, girls exhibited an average age of onset of growth spurt and age of peak height velocity at 8.57 ± 0.68 years and 10.99 ± 0.74 years, respectively, indicating that peak height velocity occurs at approximately 2.42 years after the onset of the pubertal growth spurt. Therefore, it is evident that girls experience the pubertal growth spurt at an earlier age as compared to boys, with the age of onset of growth spurt and age of peak height velocity occurring 1.60 and 1.47 years earlier in girls, respectively. Furthermore, boys exhibit a faster growth rate at the beginning of the pubertal growth spurt and undergo a steeper growth phase during this period. Specifically, girls demonstrated an average onset of growth spurt velocity and peak height velocity of 5.10 ± 0.75 and 8.32 ± 1.09 cm/year, respectively, resulting in a difference of 3.22 cm/year. Contrarily, boys displayed an average onset of growth spurt velocity and peak height velocity of 5.43 ± 0.71 and 9.61 ± 1.26 cm/year, respectively with a difference of 4.19 cm/year. Therefore, boys exhibited a 1.30-cm/year greater peak height velocity, with a specific net increase in velocity during the spurt (peak height velocity-onset of growth spurt velocity) of 0.97 cm/year higher as compared to that of girls. Regarding the duration of the pubertal growth spurt (growth spurt interval), no significant difference was observed between the boys and girls, with the growth spurt interval averaging 3.58 and 3.72 years, respectively. The boxplots in Figure 3 further illustrate the pubertal growth parameter distribution, clearly showing that, while the duration of the pubertal growth spurt is similar between boys and girls, it occurs later and is of greater magnitude among boys.

**Table 1:** Descriptive statistics of pubertal growth parameters.

	Mean	Std.Dev.	3%	5%	10%	25%	50%	75%	90%	95%	97%
Panel (a) boys											
AOGS^a^	10.17	(0.61)	9.14	9.18	9.35	9.83	10.17	10.54	10.83	11.08	11.20
APHV^b^	12.46	(0.69)	11.25	11.33	11.52	12.00	12.50	12.92	13.23	13.49	13.67
OGSV^c^	5.43	(0.71)	4.16	4.41	4.49	4.94	5.39	5.90	6.37	6.66	6.75
PHV^d^	9.61	(1.26)	7.36	7.80	7.94	8.76	9.56	10.47	11.29	11.81	11.97
GSI^e^	3.58	(0.47)	2.80	2.83	3.00	3.25	3.58	3.92	4.23	4.33	4.53
Panel (b) girls											
AOGS^a^	8.57	(0.68)	7.28	7.53	7.75	8.08	8.50	9.08	9.50	9.64	9.97
APHV^b^	10.99	(0.74)	9.83	9.83	10.00	10.50	11.00	11.50	11.95	12.31	12.52
OGSV^c^	5.10	(0.67)	4.20	4.24	4.38	4.61	4.96	5.45	5.97	6.21	6.63
PHV^d^	8.32	(1.09)	6.86	6.91	7.15	7.51	8.10	8.91	9.74	10.12	10.81
GSI^e^	3.72	(0.47)	2.83	3.03	3.08	3.42	3.75	4.08	4.25	4.39	4.47

^a^ Age of onset of growth spurt.

^b^ Age of peak height velocity.

^c^ Onset of growth spurt velocity.

^d^ Peak height velocity.

^e^ Growth spurt interval.

The correlation matrix of the three random SITAR parameters presented in Table 2 detailed the relationships between random parameters: the timing of pubertal growth spurt (tempo parameter, *β*), growth velocity (velocity parameter, *γ*), and overall height (size parameter, *α*), which were introduced in the “Data analysis” subsection of the “Methodology” section. For both boys and girls, the size parameter exhibited a positive correlation with the velocity parameter (0.33 and 0.29, respectively), suggesting that taller individuals tend to experience a larger peak height velocity during puberty. Conversely, the tempo parameter demonstrated a negative correlation with the velocity parameter (−0.43 and −0.36, respectively), implying that individuals who enter puberty later are more likely to have a smaller peak velocity. Moreover, for girls, the tempo parameter exhibited a positive correlation with the size parameter (0.18), indicating that those who experience puberty later tend to be taller in stature. This positive correlation was found to be weaker for boys (0.09).

**Table 2:** Correlations of SITAR random parameters.

	Size	Tempo	Velocity
Panel (a) boys			
Size	–	0.09	0.33
Tempo	0.09	–	−0.43
Velocity	0.33	−0.43	–
Panel (b) girls			
Size	–	0.18	0.29
Tempo	0.18	–	−0.36
Velocity	0.29	−0.36	–

The size (*α*), tempo (*β*), and velocity (*γ*) represent the three random parameters in the SITAR model, which were introduced in the “Data analysis” subsection of the “Methodology” section. Size adjusts the height of the curve; tempo adjusts the timing of the curve on the age scale and velocity scales the curve horizontally.

**Figure 4:**
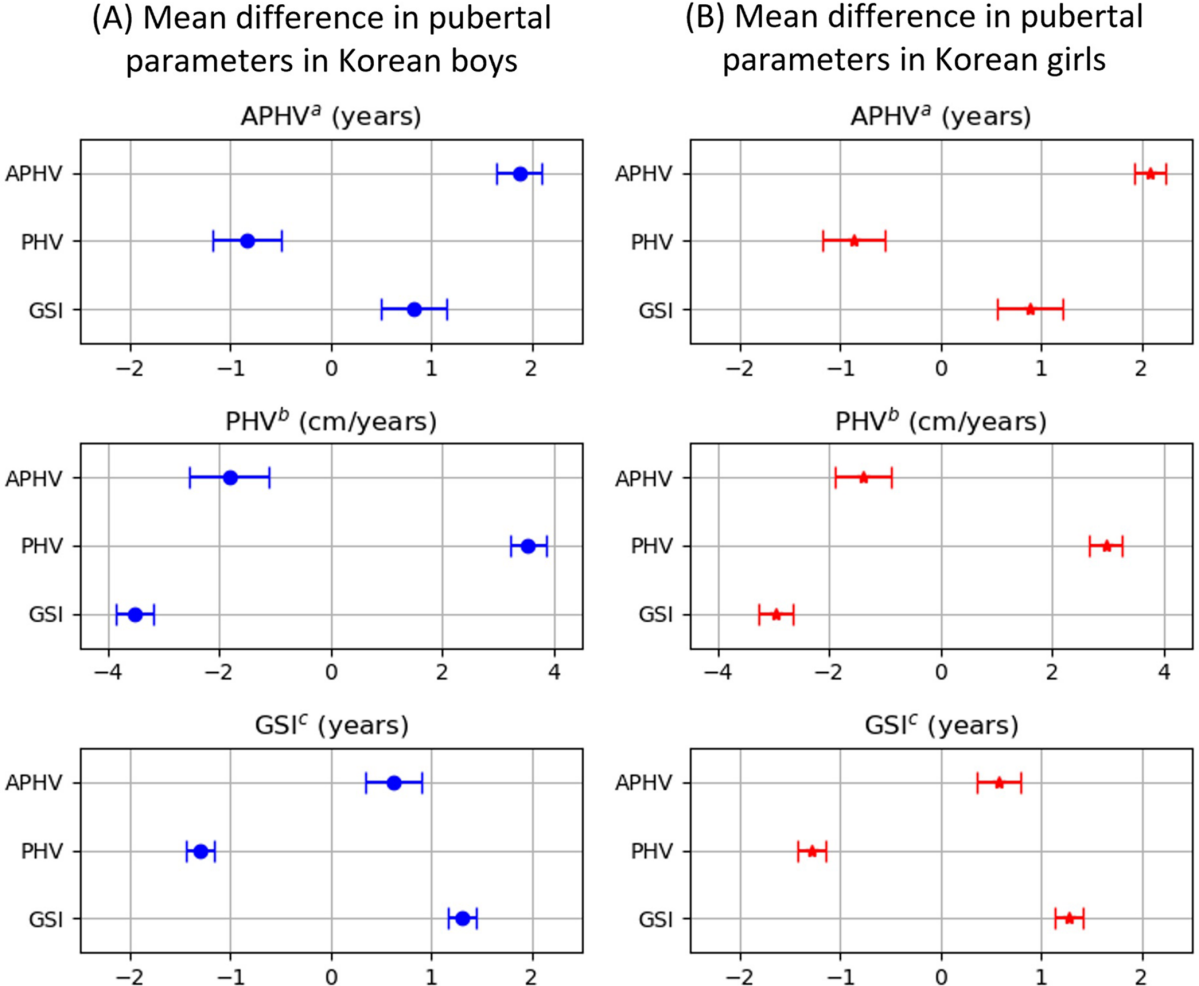
The mean differences in pubertal parameters between the top 20% and bottom 20% samples. **(A)** Mean difference in pubertal parameters in Korean boys. **(B)** Mean difference in pubertal parameters in Korean girls. ^a^APHV, age of peak height velocity. ^b^PHV, peak height velocity. ^c^GSI, growth spurt interval.

To further investigate the relationship between pubertal growth parameters, we divided the data into the top 20% and bottom 20% percentiles based on each pubertal growth parameter. Subsequently, we computed the mean difference of the other pubertal growth parameters. Table 3 segregates the samples into the top and bottom 20% percentiles based on the row variables and subsequently computes the mean difference of the column variables. For boys, the diagonal elements illustrated that the differences between the top 20% and bottom 20% percentiles for age of peak height velocity, peak height velocity, and growth spurt interval were 1.87 years, 3.53 cm/year, and 1.30 years, respectively. For girls, these differences were 2.09 years, 2.96 cm/year, and 1.28 years. Individuals with a faster age of peak height velocity exhibited a shorter growth spurt interval and a larger peak height velocity, demonstrating a negative relationship between peak height velocity and growth spurt interval. For boys, those with a faster age of peak height velocity displayed a 0.63-year shorter growth spurt interval and a 1.82 cm/year larger peak height velocity as compared to those with a slower age of peak height velocity. Furthermore, the group with a longer growth spurt interval had a 3.51-cm/year smaller peak height velocity than the group with a shorter growth spurt interval. For girls, those with a quicker age of peak height velocity had a growth spurt interval that was 0.58 years shorter and a peak height velocity that was 1.39 cm/year greater than those with a slower age of peak height velocity. Moreover, those with a longer growth spurt interval had a peak height velocity that was 2.96 cm/year smaller than those with a shorter growth spurt interval. All differences were significant at the 5% significance level, with most comparisons reaching a *p*-value of <0.0001. The mean differences and confidence intervals are visually depicted in Figure 4.

**Table 3:** The mean differences in pubertal parameters (column variables) between the top 20% and bottom 20% samples (based on the row variables).

	APHV^a^	PHV^b^	GSI^c^
Panel (a) boys			
APHV^a^	1.87*	−1.82*	0.63*
PHV^b^	−0.84*	3.53*	−1.30*
GSI^c^	0.83*	−3.51*	1.30*
Panel (b) girls			
APHV^a^	2.09*	−1.39*	0.58*
PHV^b^	−0.87*	2.96*	−1.28*
GSI^c^	0.88*	−2.96*	1.28*

^a^ Age of peak height velocity.

^b^ Peak height velocity.

^c^ Growth spurt interval.

* *P* < 0.001.

## Discussion

The pubertal growth spurt plays a pivotal role in the overall growth process (23). In the present study, we employed longitudinal height growth data to comprehensively examine the characteristics of the pubertal growth spurt among Korean boys and girls. During pubertal growth, girls typically displayed an earlier age of onset of growth spurt (10.17 and 8.57 years for boys and girls, respectively) and age of peak height velocity (12.46 and 10.99 years for boys and girls, respectively), along with lower onset of growth spurt velocity (5.49 and 5.10 cm/year for boys and girls, respectively) and peak height velocity (9.61 and 8.32 cm/year for boys and girls, respectively), as well as longer growth spurt interval (3.58 and 3.72 years for boys and girls, respectively) than boys. Furthermore, our analysis revealed significant associations among the pubertal growth parameters. Specifically, we found that earlier puberty onset, in both boys and girls, is linked to a shorter growth spurt interval (0.63 and 0.58 years for boys and girls, respectively) and a larger peak height velocity (1.82 and 1.39 cm/year for boys and girls, respectively).

The findings can be compared with results from previous studies on pubertal growth. According to the 2017 Korean National Growth Charts (KNGC2017), the age of peak height velocity was 12.9 years for Korean boys and 10.3 years for Korean girls, corresponding to the 50th to 75th percentile range for boys and the 10th to 25th percentile range for girls compared to our analyzed age of peak height velocity data (24). In KNGC2017, peak height velocity was reported as 7.7 and 6.9 cm/year for boys and girls, respectively. However, corresponding to our analyzed peak height velocity data, both boys and girls fall into the 3rd to 5th percentile range. This significant difference between our research data and KNGC2017 may be due to the use of cross-sectional data in KNGC2017, which qualitatively differs from our longitudinal study data. To address this, we compared our data with a study in Kangwha Province, Korea, also using longitudinal data (25). In this study, the age of peak height velocity was 12 and 10 years for boys and girls, respectively, and peak height velocity was 8.62 and 7.07 cm/year for boys and girls, respectively. Despite the challenge of comparing yearly intervals in Kangwha with our monthly analyzed age of peak height velocity data, peak height velocity values showed significant differences. For peak height velocity, corresponding to our analyzed data, boys fell into to 10th to 25th percentile range and girls fell into the 5th to 10th percentile range. Both KNGC2017 and Kangwha datasets indicated lower peak height velocity compared to our research findings. This difference may be attributed to the timing of the study and regional characteristics. KNGC2017 used data from 2005, while the Kangwha study used data from 1986 to 1999, over 10 to 20 years before our study data (2013 to 2023). Additionally, Kangwha, a rural area, showed differences compared to Gwangmyeong City, the focus of our study. The data suggest that the current generation in metropolitan areas experiences accelerated peak height velocity compared to previous generations. However, the influence of the SITAR model used in our study cannot be excluded, necessitating ongoing analysis to support our findings. In comparison to previous international studies, a longitudinal study involving Taiwanese children revealed age of peak height velocity estimates of 12.5 and 10.5 years for boys and girls, respectively, which were similar to our study findings (26). Longitudinal studies conducted on Western populations generally demonstrate a later age of peak height velocity as compared to studies involving Asian populations (27–29).

Several previous studies have reported a global trend towards an earlier average onset of puberty (30–32). To analyze the earlier onset of puberty objectively, various countries are employing the age of peak height velocity as a standardized measure (33, 34). Danish boys and girls born in the 1930s had estimated age of peak height velocities of 14.5 and 12.5 years, respectively, which declined to 14.2 and 12.0 years, respectively, for those born in the 1960s (35). In the Gothenburg BMI Epidemiology Cohort (Sweden), the estimated age of peak height velocity remained stable among boys born in the 1940s and 1950s at 14.2 years, which gradually declined to 13.7 years among those born in 1996 (11). In comparison, our study analyzed a delayed age of peak height velocity as compared to that obtained using the KNGC2017 data, but drawing meaningful conclusions is challenging due to the differences in data characteristics and the lack of analysis using the same SITAR model in the KNGC2017. However, although this study utilizes earlier data compared to the KNGC2017 dataset, T.J. Cole and H. Mori analyzed secular trends in height growth using anthropometric data collected from nationwide cross-sectional surveys in Korea between 1965 and 2005, similar to the KNGC2017 data collection method, employing the SITAR method (36). While their study focused on chronological cross-sectional variations using SITAR, our study analyzes contemporary longitudinal data using the same approach. Therefore, a direct comparison between the two studies is challenging. Nevertheless, they revealed that over the 40-year period, the age of onset of the growth spurt advanced and peak height velocity increased in both sexes; however, as the growth spurt interval decreased, suggests that while the acceleration and steepening of the growth spurt occurred, these changes were not identified as primary determinants of the height increase during this period.

Also, since SITAR model is highly advantageous for analyzing pubertal parameters because it fits all individuals into a single model, uses a flexible cubic spline for the mean curve, and incorporates subject-specific random effects for relative size and growth intensity (37, 38). Consequently, applying SITAR in puberty-related studies allows for the comparison of pubertal parameters across different disease groups and establishes reference data that can lay the groundwork for a variety of research endeavors.

This study had several limitations. Firstly, the age range of the included students was limited to 7–18 years. As mentioned earlier, given the ongoing trend of advancing puberty onset age and age of peak height velocity, it would be beneficial to enroll participants with a wider pre-pubertal age range. Secondly, obtaining information on the final adult height of the participants included in the analysis would enable the exploration of the associations between the growth parameters utilized in the SITAR model and final height. Thirdly, while our study primarily sourced data from the non-metropolitan region of Gwangmyeong City in Gyeonggi Province, potentially limiting its representation of the general Korean populace, its robustness remains notable. In 2023, Gyeonggi Province, including Gwangmyeong City, had an average annual income of approximately 50,000,000 Korean Won (KRW), higher than the national average of 48,425,988 KRW, largely due to Gyeonggi's proximity to Seoul and its active economic environment. However, the Seoul-Gyeonggi region importantly includes 44.8% of the national population aged 0–19, indicating that despite economic differences, it still represents a significant portion of South Korea's demographic landscape. To enhance data representativeness, we have expanded data collection to additional regions beyond Gwangmyeong, covering a broader range of age groups since 2015, aiming to better reflect the Korean child and adolescent population in our research.

However, the present study holds several strengths. This study applied the SITAR model to assess pubertal growth in Korean children and adolescents using recent, large-scale, longitudinal data. The study analyzed the characteristics of pubertal growth spurts in Korean children and adolescents by examining tempo, velocity, and size, which offers the advantage of more accurately reflecting individual growth patterns due to the longitudinal nature of the data. Additionally, this approach allows for more detailed analyses, such as extending data collection across broader age ranges and performing regression analyses to uncover relationships among growth parameters. Furthermore, in future studies, by incorporating additional variables such as parental height, obesity or underweight status, and underlying health conditions, this approach can be utilized to further analyze how these factors influence each parameter of the pubertal growth spurt.

## Conclusion

In conclusion, by utilizing longitudinal height growth data and applying the SITAR methodology to investigate the features of pubertal growth spurts in Korean children and adolescents, this study has established reference height velocity curves for both sexes throughout puberty and estimated pubertal growth parameters.

## Data Availability

The datasets presented in this article are not readily available because the authors do not have ownership rights to distribute or share the data. Requests to access the datasets should be directed to Jaehyuk Sung, sjh@gpcohort.com.
